# Case of Uterine Hemorrhage, Terminating Fatally

**Published:** 1828-02

**Authors:** C. Waller


### UTERINE HEMORRHAGE.

### *Case of Uterine Hemorrhage, terminating fatally.*

By C. Waller, Esq.

The following case of fatal hemorrhage, to which I was summoned on the 5th January, is, I think, one of sufficient importance to be admitted on the pages of your Journal, as tending to strengthen a position which has hitherto been warmly opposed,—viz. that cases may and do occur, wherein the patient sinks from exhaustion, although the flooding has been permanently stopped by the tonic contraction of the uterus. Not having been present till near its termination, I must refer you to the inclosed letter, which was most obligingly furnished me by Mr. Birch.

*29, Charlotte-street, Bloomsbury-square;*

*January 6th*, 1828.

Dear Sir,—With reference to the case in which you were good enough to give me your assistance yesterday, I am very happy to make the statement you desire: and I beg you will make whatever use of it you may deem proper.

I was called to the poor woman by the gentleman in attendance upon her from the Finsbury Midwifery Institution, about half-past seven in the morning. I found her powers were then very much sunk by hemorrhage from the uterus, which had taken place at intervals for several days, and which had become gradually more profuse: her countenance was exsanguineous, her pulse very feeble and rapid; her extremities cold; and she was unwilling to move, to speak, or to be spoken to. There were no labour pains; the os uteri was about an inch and a half in diameter, thin and dilatable; the placenta was attached over it to every part of its circumference. Without much difficulty, I passed my hand through the os uteri, peeled off a part of the placenta from the surface of the uterus, passed my hand into its cavity, and brought down the feet of the child. There was considerable difficulty in extracting the head, which prevented the operation from being speedily accomplished. The pelvis was undoubtedly small, for the woman had given birth to five or six stillborn children, after tedious labours. Very little blood was lost during the turning of the child, and subsequent extraction of the placenta; but, little as it was, it and the operation proved sufficient to render the woman's state still more alarming: the extremities became colder, the pulse more feeble, and extreme restlessness, convulsive movements, and insensibility alternated with each other.

I sent to request my colleague, Dr. Conquest, would bring his apparatus for transfusion; but, unfortunately, when he arrived, it was found defective, the pipe was lost, and it was useless. In this dilemma, you politely consented to lend us your syringe and your assistance; but so much time had unavoidably been lost before your arrival, (nine A.M.) and so little had the most active employment of the ordinary remedies, brandy and laudanum, been able to effect for this poor woman, that, at the time you entered her miserable apartment, I certainly considered her in articulo mortis: the pulse was not to be felt at the wrist; the heart's action was a mere flutter; the extremities were perfectly cold; *the respiration had sunk to an occasional sigh;* the power of deglutition was gone; and she lay perfectly unconscious and motionless. Twice, sir, you know we injected a quantity of blood, (which you will be able to estimate, from knowing the capacity of your syringe,) into the median vein, without producing any apparent change in the woman's condition. Indeed, I am much mistaken if she were not already dead when the second injection was made.

It may be satisfactory to you, sir, if I (who do not profess to be an enthusiastic admirer of transfusion,) state my conviction that it would be unjust to draw any unfavorable opinion of it from this case, which at most can only tend to prove that it has not the power of raising to life the dying and the dead; the contrary of which position, if I mistake not, its most staunch friends have not ventured to advocate.

I am, sir, your most obedient humble servant,

WM. BIRCH.

C. WALLER, Esq. Bartholomew Close.

The quantity of blood injected was very trifling: the person from whom it was taken did not bleed very freely, and the urgency of the case demanded that no time should be lost. I therefore contented myself with half a syringe full, (about one ounce,) and repeated this as quickly as possible; but the patient had ceased to breathe before the syringe was introduced the second time: and indeed my firm conviction is, that the circulation was too far reduced to allow of the passage of the first ounce into the auricle. Strongly does this argue the necessity of our being upon the watch on these occasions, and not deferring the operation until the patient has arrived at the last gasp. The experiments of Dr. BLUNDELL prove that, although blood be injected *immediately* after the cessation of respiration, yet still the animal will not be recovered; and, in the case just related, although it could not perhaps be said to have completely ceased, yet it was performed very imperfectly; nor could I discover the action of the heart through the thoracic parietes.

The time *necessarily* lost before the operation was attempted, is much to be regretted; for I am thoroughly persuaded in my own mind, from what I have seen of transfusion, that, had the apparatus been perfect at the time Dr. Conquest arrived at the house, the transfused blood would have been impelled through the vessels, and the unfortunate female would have been at this moment another living example of the efficacy of the practice.

Let it not, however, be supposed that we are visionary enough to expect that success will *invariably* crown our exertions. Transfusion should be judged of in comparison with the other operations of surgery; and, should it be found to succeed equally with them, ought, in my opinion, to be as generally received. It should be borne in mind, too, that, in the present state of our knowledge, we only employ it in the most desperate cases.

*Bartholomew Close; January 8th,* 1828.

---

SMALL-POX.

